# Beyond the Obstructive Paradigm: Unveiling the Complex Landscape of Nonobstructive Coronary Artery Disease

**DOI:** 10.3390/jcm13164613

**Published:** 2024-08-07

**Authors:** Andreea Tudurachi, Larisa Anghel, Bogdan-Sorin Tudurachi, Alexandra Zăvoi, Alexandr Ceasovschih, Radu Andy Sascău, Cristian Stătescu

**Affiliations:** 1 Cardiology Department, Cardiovascular Diseases Institute “Prof. Dr. George I. M. Georgescu”, 700503 Iași, Romania; leonteandreea32@gmail.com (A.T.); alexandra.zavoi@gmail.com (A.Z.); radu.sascau@umfiasi.ro (R.A.S.); cristian.statescu@umfiasi.ro (C.S.); 2 Internal Medicine Department, “Grigore T. Popa” University of Medicine and Pharmacy, 700503 Iași, Romania; alexandr.ceasovschih@yahoo.com; 3 “St. Spiridon” Clinical Emergency Hospital, Independence Boulevard No. 1, 700111 Iasi, Romania

**Keywords:** nonobstructive coronary artery disease, ANOCA, INOCA, microvascular dysfunction, immunoinflammatory pathways, multimodal imaging

## Abstract

Traditionally focused on obstructive atherosclerosis, contemporary research indicates that up to 70% of patients undergoing coronary angiography for angina and ischemic symptoms do not exhibit significant stenoses. Nonobstructive coronary artery disease (CAD) has emerged as a prevalent phenotype among these patients. This review emphasizes the emerging understanding that nonobstructive coronary artery disease, encompassing conditions such as ANOCA (Angina with No Obstructive Coronary Artery Disease), INOCA (Ischemia with No Obstructive Coronary Artery Disease), and MINOCA (Myocardial Infarction with No Obstructive Coronary Arteries), represents the most prevalent phenotype in cardiac patients. It delves into the complex pathophysiology underlying these conditions, focusing on microvascular dysfunction and coronary vasoreactivity, which contribute to myocardial ischemia despite the absence of significant coronary obstructions. Additionally, the review critically examines the limitations of current treatments which primarily target obstructive lesions and underscores the necessity for tailored therapies that address the specific microvascular and immunoinflammatory pathways involved in nonobstructive CAD. The main focus of this review is to advocate for a shift in diagnostic and therapeutic strategies to better identify and manage this widely prevalent yet under-recognized subset of CAD.

## 1. Introduction

Globally, angina pectoris affects about 100 million individuals and is the predominant manifestation of myocardial ischemia. For more than a hundred years, the main clinical characteristic was believed to be obstructive atherosclerosis affecting the larger coronary arteries. A significant number of patients (up to 70%) undergoing coronary angiography for angina and signs of myocardial ischemia do not have obstructive coronary arteries, yet they exhibit detectable ischemia [1,2]. In recent years, the terminology and classification of coronary artery diseases have evolved to better describe various clinical presentations. Up until 2020, the terms INOCA (Ischemia with Non-Obstructive Coronary Arteries) and MINOCA (Myocardial Infarction with Non-Obstructive Coronary Arteries) were well-established in the literature [1,3]. INOCA typically involves the presence of documented ischemia through stress testing or other diagnostic modalities, despite the absence of significant coronary artery blockages, typically resulting from microvascular dysfunction or coronary artery spasm [1,3]. MINOCA is defined by myocardial infarction evidenced by elevated cardiac biomarkers, occurring in the absence of significant coronary artery obstructions, with etiologies ranging from atherosclerotic plaque events to non-atherosclerotic causes like coronary artery dissection [1,3]. However, a new term, ANOCA (Angina with Non-Obstructive Coronary Arteries), has emerged in subsequent years to further refine the classification of these conditions [4,5]. Nonobstructive CAD represents an important paradigm shift in understanding cardiovascular pathophysiology. While traditionally underrecognized, it is now clear that this condition significantly impacts patient outcomes and requires dedicated research and clinical focus. The inclusion of ANOCA provides a more comprehensive framework for understanding and managing patients presenting with angina without significant coronary artery obstruction, thereby enhancing the complexity of non-obstructive coronary artery disease classification.

**Figure 1:**
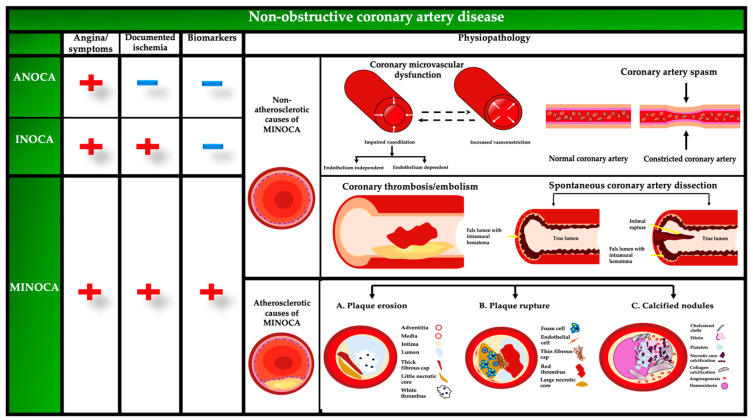
Physiopathology of non-obstructive coronary artery disease. ANOCA, angina with no obstructive coronary artery disease; INOCA, ischemia with no obstructive coronary artery disease; MINOCA, myocardial infarction with no obstructive coronary arteries.

Individuals in this category are referred to as having ANOCA, INOCA, or MINOCA. ANOCA is characterized by angina symptoms in the absence of significant coronary artery obstruction, often due to coronary microvascular dysfunction or coronary artery spasm [4]. The complexity and diversity of non-obstructive coronary artery diseases underscore the critical need for comprehensive diagnostic approaches to effectively identify and manage these conditions. Microvascular causes of non-obstructive CAD may be classified based on their pathophysiology into structural and functional processes, as well as myocardial variables that contribute to microvascular function and result in a decrease in blood flow to the heart muscle [6] (Figure 1).

Thus, the aim of this narrative review is to better understand the three phenotypes of nonobstructive CAD, as to what mechanisms underly each of them, which clinical approach is needed for each of them, and which therapeutic measures may be offered tailored to the unique characteristics of these conditions. 

## 2. Epidemiological Data

INOCA predominantly affects middle-aged to older adults, with a higher prevalence in females, often associated with microvascular dysfunction and vasospasm. It affects approximately 47% of women and 30% of men with suspected cardiac chest pain, reflecting a significant gender disparity [7]. 

MINOCA occurs in younger to middle-aged individuals and also shows a higher incidence in women. It can involve altered coagulation and non-atherosclerotic causes of myocardial infarction, with an incidence rate ranging from 2.9% to 13.8%, predominantly in non-ST elevation myocardial infarction (NSTEMI) cases [8].

ANOCA is a recently defined condition affecting a broad age range, with a notable female predominance. Patients presenting with angina symptoms but without obstructive coronary artery disease often require further investigation to identify potential microvascular dysfunction or other non-obstructive causes of ischemia [9]. 

Patients with angina and non-obstructive coronary artery disease (CAD) often have a lower prevalence of traditional cardiovascular risk factors, such as diabetes mellitus, compared to those with obstructive CAD. However, smoking is significantly associated with endothelial dysfunction, leading to impaired coronary artery dilation. Additionally, comorbidities such as hypertension, insulin resistance, hyperlipidemia, obesity, menopause, and chronic autoimmune inflammatory disorders are consistently linked with coronary microvascular dysfunction [1]. Age was the only variable independently associated with microvascular dysfunction, as reported by Sara et al. [10]. Furthermore, another extensive study by Kanaji Y et al. identified various risk factors for microvascular dysfunction in ANOCA. Specifically, age was a risk factor for both endothelial-dependent and independent microvascular dysfunction, female sex was a risk factor for only endothelial-independent microvascular dysfunction (influenced by adenosine), and diabetes mellitus was a risk factor for endothelial-dependent microvascular dysfunction [11]. Studies have shown that men and women have a similar occurrence of coronary vascular dysfunction. Nevertheless, it is likely that males have a higher incidence of epicardial spasm, and a lower incidence of microvascular spasm compared to women [12]. Asian individuals seem to have a higher susceptibility to vasospastic angina compared to Caucasians [10,13]. East Asian individuals often have diffused and multi-vascular coronary artery spasms (CAS), while Caucasians tend to exhibit localized CAS.

## 3. Mechanistic Insights

The specific pathophysiological processes responsible for MINOCA/INOCA/ANOCA are not yet well understood, but the primary causes may be categorized as either atherosclerotic or non-atherosclerotic. In studies on MINOCA, atherosclerotic causes include plaque disruption, whereas non-atherosclerotic causes include vasospasm, coronary microvascular dysfunction, coronary thrombosis/embolism, and spontaneous coronary artery dissection [14]. The main mechanisms of INOCA are microvascular dysfunction and epicardial coronary artery spasm [1,3]. In ANOCA, there is no evidence of microcirculatory blockage or chronic coronary spasm, unlike in MINOCA and the mechanisms include coronary microvascular dysfunction, epicardial coronary artery spasm, and a combination of both [1] (Table 1).

The coronary arterial bed is divided into three compartments: epicardial coronary arteries (500 μm to 5 mm in diameter), pre-arterioles (0.1–0.5 mm), and intramyocardial arterioles (<0.1 mm). Epicardial arteries function as conduits and do not impede blood flow when unobstructed, while pre-arterioles and intramyocardial arterioles are critical for regulating coronary blood flow. The epicardial arteries account for only 10% of the coronary circulation volume, with the microcirculation making up the remaining 90% and contributing more than 70% of the coronary system’s resistance under normal conditions. Intramyocardial arterioles have the greatest resistance and adjust blood flow based on myocardial oxygen demand through autoregulation. In healthy individuals, increased myocardial metabolic demand leads to arteriolar dilation and a significant increase in coronary blood flow, up to five times the normal level [1]. 

**Figure 2:**
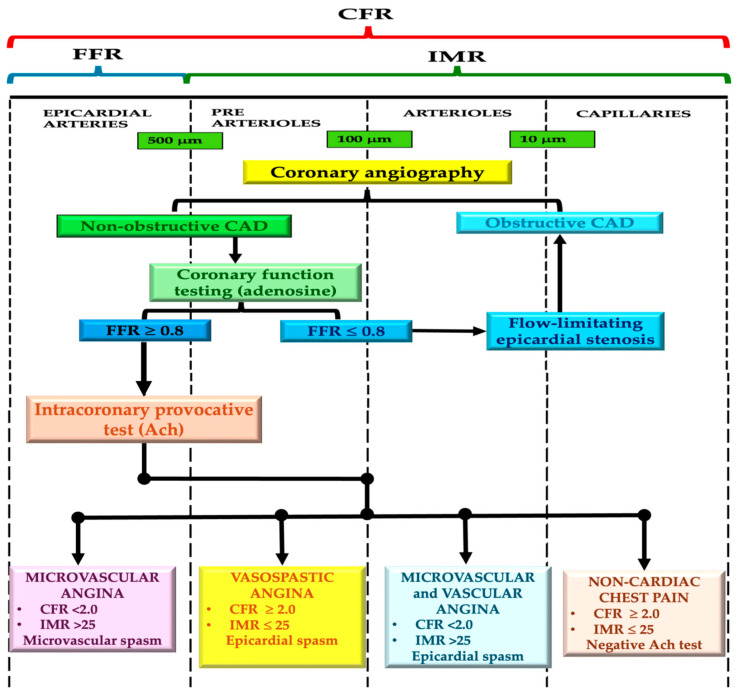
Diagnostic algorithm for invasive functional evaluation in patients with non-obstructive coronary artery disease. Ach—acetylcholine; CAD—coronary artery disease; CFR—coronary flow reserve; FFR—fractional flow reserve; IMR—index of microvascular resistance. Adapted from [1].

Although there are many noninvasive ways to evaluate ischemia in individuals with non-obstructive CAD, an invasive and systematic examination is critical for a comprehensive diagnosis of these patients and focused therapeutic management. Invasive testing provides the ability to distinguish between different endotypes, enabling the delivery of tailored and optimal therapy and management for patients with MINOCA/INOCA/ANOCA. Coronary angiography is required for all patients displaying signs and symptoms of ischemia in order to rule out the possibility of an epicardial illness. Therefore, if the angiography does not reveal any obstructive CAD, the patient may proceed with comprehensive invasive testing to diagnose any structural or functional abnormalities in the coronary artery during the same operation (Figure 2) [20]. A comprehensive assessment using coronary flow reserve (CFR) and microvascular resistance, such as invasive CFR and index of microvascular resistance (IMR), distinguishes between various pathophysiological mechanisms (Figure 2).

Invasive CFR can be measured using Doppler flow velocity or thermodilution mean transit time, with prognostic cutoff values set at 2.0 for thermodilution-based CFR (tCFR) and 2.5 for Doppler-based CFR (dCFR). IMR values ≥ 25 suggest endothelial-independent dysfunction, while hyperemic microvascular resistance (hMR) values > 2.4 indicate abnormal microcirculation. Steady-state hyperemia is typically induced by intravenous or intracoronary adenosine administration. Abnormal FFR is defined as ≤0.80 or a non-hyperemic pressure ratio ≤ 0.89, indicating flow-limiting obstructive CAD [1,4].

Comprehensive assessment enables the identification of endothelium-independent CMD using CFR and IMR, evaluation of endothelium-dependent CMD by assessing microvascular response to ACh, and detection of low-grade stenoses using FFR.

### 3.1. Coronary Plaque Disruption 

Coronary plaque disruption encompasses plaque rupture, plaque erosion, and calcified nodules. Plaque rupture is caused by defects in the fibrous cap, exposing the thrombogenic core due to vascular smooth muscle cells (VSMCs) depletion and macrophage invasion, and is prevalent in the elderly. Plaque erosion, often found in younger individuals, females, and smokers, involves endothelial cell apoptosis and detachment from the extracellular matrix (ECM), leading to blood clots on plaques with low lipid content. Calcified nodules, although rare, create uneven arterial lumen boundaries and may increase thrombosis risk due to impaired endothelial cells. Variations in pro-inflammatory biomarkers such as soluble vascular cell adhesion molecule-1 (sVCAM-1) and Chemokine (C-C motif) ligand 21 (CCL-21) in MINOCA patients suggest that plaque structure changes and microcirculatory alterations contribute to atherosclerosis progression. Starting statin therapy is strongly recommended, while other heart-protective medications should be individualized [21,22,23,24,25,26].

### 3.2. Coronary Artery Spasm

**Figure 3:**
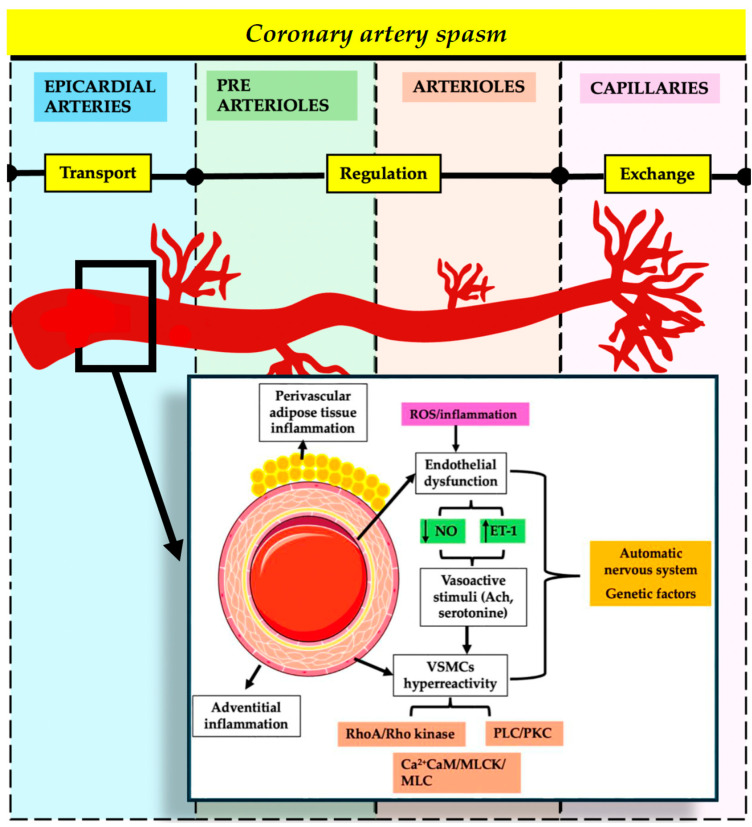
Physiopathology of coronary artery spasm. Ach—acetylcholine; CaM—calmodulin; ET-1—endothelin-1; NO—nitric oxide; MLC—myosin light chain; MLCK—myosin light chain kinase; PLC—phospholipase C; PKC—protein kinase C; ROS—reactive oxygen species; RhoA—Ras homolog gene member A; VSMCs—vascular smooth muscle cells.

Coronary artery spasm (CAS) involves severe narrowing (>90%) of the epicardial coronary artery, leading to chest discomfort and ECG abnormalities, often triggered by various stimuli. The pathophysiology of CAS is complex and yet not completely understood (Figure 3). The mechanisms of CAS include hyperreactivity of VSMCs and endothelial dysfunction, involving pathways such as Ca2+/calmodulin-activated myosin light chain kinase and Rho-kinase. Inflammation, oxidative stress, and genetic factors also contribute to CAS, with inflammatory markers such as high-sensitive C protein reaction (hs-CRP) and interleukin-6 (IL-6) playing significant roles. Smoking is related mainly to epicardial spasm, and not as much to myocardial microvascular blood flow [11]. Management of INOCA patients includes addressing potential harms from prolonged nitrate use and utilizing invasive physiological testing to guide treatment, as supported by the CorMicA study which showed improved outcomes with tailored medical therapy. Smoking cessation and avoiding vasoconstrictive drugs are crucial preventive measures, especially for young women, as smoking significantly impacts endothelial function and NO availability [27,28,29,30].

Recent research indicates that patients with premature (M)INOCA have distinct risk factor profiles compared to those with obstructive CAD, with atypical risk factors such as migraines, preeclampsia, depression, and anxiety contributing to (M)INOCA. Psychological stress is more likely to cause endothelial dysfunction and vasomotor issues in young women than in men. Additionally, factors like alcohol withdrawal, everyday stress, certain medications, and cold exposure can trigger anginal episodes. Long-term depression and chronic inflammation are linked to abnormal vascular reactivity, increasing the risk of (M)INOCA, particularly in women. Effective management of INOCA involves lifestyle changes, risk factor management, and the use of medications such as calcium-channel blockers, with short-acting nitrates as secondary options and careful avoidance of β-blockers [31,32,33].

### 3.3. Coronary Microvascular Dysfunction

**Figure 4:**
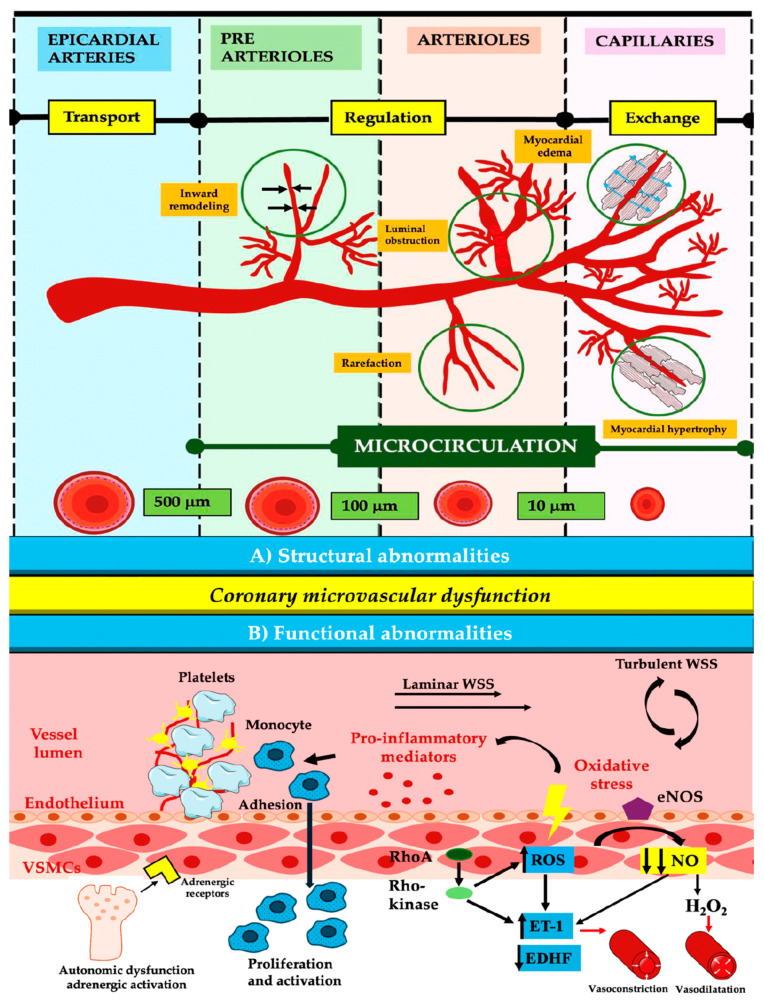
Physiopathology of coronary microvascular dysfunction. ET-1—endothelin-1; EDHF—endothelium-dependent hyperpolarization factors; NO—nitric oxide; eNOS—endothelial NO synthase; H2O2—hydrogen peroxide; ROS—reactive oxygen species; RhoA—Ras homolog gene member A; VSMCs—vascular smooth muscle cells; WSS—wall shear stress.

Coronary microvascular dysfunction affects the small blood vessels in the coronary arteries, leading to impaired blood flow and abnormal resistance. Additionally, as shown in Table 1, coronary microvascular dysfunction is present in ANOCA and INOCA. The primary mechanisms involve increased vasoconstriction through the phosphorylation of myosin light-chain by Rho-kinase and the activation of the RhoA/Rho-kinase pathway, which leads to the production of reactive oxygen species (ROS), endothelin-1 (ET-1), and proinflammatory molecules (Figure 4). 

The perfusion anomalies observed in two-thirds of female MINOCA patients undergoing stress cardiac CMR were identified as CMD. Furthermore, of the 80 MINOCA patients who had ACh testing within 48 h after admission, 46% received a positive result, and 35 percent of the positive cases developed microvascular spasms. Both endothelium-dependent and endothelium-independent pathways contribute to impaired vasodilation, with factors like nitric oxide (NO), prostaglandins, and endothelium-dependent hyperpolarization factor (EDHF) playing crucial roles. Endothelial dysfunction, observed in about 50% of INOCA patients, is linked to worsening symptoms and increased adverse outcomes [5,17,34,35]. Inflammation-induced activation of endothelial cells leads to increased production of ROS and adhesion molecules, causing platelet and leukocyte adhesion. NOx isoforms and mitochondria regulate ROS formation, with NOx activation enhancing ROS production through p66Shc phosphorylation. This process impairs vasodilation by converting NO into peroxynitrite radicals. Epigenetic modifications disrupt the balance between oxidants and antioxidants, increasing ROS and pro-inflammatory cytokines. This results in endothelial cell activation, loss of endothelial barrier function, and damage to coronary microvascular endothelial cells, further exacerbated by pathways involving NLRP3, caspase-1, interleukin 1b (IL-1b), and interleukin-18 (IL-18). These mechanisms collectively contribute to CMD and related cardiovascular issues [36,37,38,39,40].

The Spanish ENDOCOR registry found that patients with endothelial dysfunction had more severe angina symptoms and were more likely to experience angina with moderate exertion and adverse cardiac events within one year. Despite the use of acetychcoline (ACh) testing to guide therapy, optimal treatment was not consistently achieved, and vasodilator therapy did not significantly improve clinical outcomes [17].

In patients without obstructive CAD, the soluble urokinase plasminogen activator receptor (suPAR) and higher IL-6 levels are linked to CMD and worse outcomes. Inflammation is a critical factor in MINOCA, contributing to plaque rupture, myocardial damage, and ischemia/reperfusion injury, leading to ROS production and cell death. Wall shear stress influences endothelial cell inflammatory responses, affecting NO production and promoting atherosclerosis when low. Aging and oxidative stress impair microvascular endothelial function, reducing NO and increasing inflammatory cytokines, contributing to CMD. Platelet adhesion to the endothelium further exacerbates inflammation and vascular damage. Chronic autonomic dysfunction, structural abnormalities in microvessels, and external compression due to various conditions also contribute to CMD. CMD predicts adverse outcomes in patients with non-obstructive CAD, emphasizing the need for further functional examinations for risk classification. While trials like ORBITA and ISCHEMIA focus on stable CAD treatment, the role of CMD in ongoing ischemia post-revascularization highlights the need for understanding coronary microvascular physiology [3,7,41,42,43,44,45].

## 4. Clinical Implications

Current therapy for CMD focuses on reducing risk factors and managing disease processes [1]. 

Sodium-glucose transport Protein 2 (SGLT2) inhibitors, known for their positive impact on the heart, have been shown to enhance coronary microvascular function in mice, decrease inflammation-induced ROS, and increase endothelial NO availability [46].

Statins, known for their antioxidant and anti-inflammatory properties, have been found to alleviate impaired coronary artery function in pigs and enhance coronary flow reserve (CFR) in non-obstructive CAD patients. Additionally, studies have shown that statins enhance CFR in people who do not have obstructive CAD. A randomized trial assessing a specific index of microvascular resistance (IMR) in women with INOCA found no improvement in microvascular function after 6 months of therapy [47,48]. 

HsCRP is considered a notable predictor of CMD and a risk factor for coronary artery stenosis. Tong et al. discovered a correlation between hsCRP levels and peak troponin levels, suggesting a link between inflammation and myocardial damage or infarction. 

IL-6, a risk marker for atherothrombotic events, is elevated in the plasma of individuals with non-obstructive CAD, including coronary microvascular dysfunction and coronary artery spasm. Thus, IL-6 may possess the potential to predict the onset and etiology of non-obstructive myocardial infarction. A study on tocilizumab, an IL-6 receptor antagonist, found no effect on coronary microvascular function in patients with NSTEMI during hospitalization or after six months [49]. 

Cheng et al. found that inhibiting myeloperoxidase (MPO) reduced inflammation-induced endothelial dysfunction in mouse models of vascular inflammation and atherosclerosis, highlighting its role in oxidative stress and inflammatory reactions [50]. For the first time, the Lasso study conducted a comprehensive investigation of a wide range of cardiovascular biomarkers during the stable period after MINOCA. The primary finding was a higher level of proinflammatory activity in patients with MINOCA compared to both MI-CAD and the control group [51].

Giving tumor necrosis factor inhibitors to people with psoriasis greatly improved the function of their coronary microvascular system, as measured by coronary flow reserve, and lowered levels of biomarkers of systemic inflammation. Other possible approaches to reducing vascular inflammation include focusing on cholesterol metabolism, fatty acid mediators, and the autophagy-lysosome pathway [35,52]. 

IL-1b has a critical role in initiating IL-6 signaling, which necessitates the use of anti-inflammatory treatments in CMD. Low-dose methotrexate has been ineffective in decreasing inflammation in people with CAD, although colchicine is now under investigation for this purpose. The potential of COLCOT studies and other modulators of interleukin-1 (IL-1), IL-6, and NLRP3 inflammasome is promising [49].

Cytokines exhibit significant potential as diagnostic tools and serve as biomarkers for various disorders. Within these, visfatin, placental growth factor (PlGF), and fractalkine (CX3CL1) can immediately initiate issues in the blood vessels, inflammation, and the formation of new blood vessels by activating a cellular signaling pathway known as nuclear factor Kappa B (NF-κB). Although cytokines have regular functions in the body, they become too active in the processes that lead to MINOCA [53].

Researchers looked at changes in time and biomarker levels in MINOCA patients and found that these patients had higher levels of early inflammatory activity during the acute phase, more temporary effects of myocardial damage, and faster recovery than MI-CAD patients. C-reactive protein (CRP) was shown to be a reliable predictor of both all-cause mortality and MACE in patients with MINOCA. Moreover, they discovered several biomarkers that possess discriminative utility in distinguishing between MINOCA and MI-CAD. When comparing MINOCA with MI-CAD, it was shown that CRP, tumor necrosis factor (TNF)-related activation-induced cytokine (TRANCE), tissue-type plasminogen activator (t-PA), and MPO were able to differentiate between the two conditions. Other studies show discrepancies in hs-CRP/CRP concentrations between patients with MINOCA and MI-CAD, possibly due to a lack of adjusted comparisons or variations in blood collection methods [51,54]. 

Rho-kinase activity in circulating neutrophils can serve as a valuable biomarker for coronary spasms, aiding diagnosis and evaluating disease activity and treatment effectiveness. Daily fluctuations in Rho-kinase activity, with the highest level in early mornings, are linked to changes in baseline constriction of coronary arteries and their response to blood vessel diameter changes. Using both the Japanese Coronary Spasm Association (JCSA) risk score and Rho-kinase activity significantly enhanced the process of categorizing the risk of vasospastic angina (VSA) patients in comparison to using either one of them alone. Additionally, the assessment of Rho-kinase activity in circulating leucocytes might be valuable for determining the prognosis of patients with VSA. Recent studies have shown that individuals with microvascular spasms had considerably greater levels of serotonin in their plasma compared to control subjects. These results indicate that the quantity of serotonin in the plasma might be a new biomarker for predicting latent microvascular spasms and distinguishing it from epicardial CAS [55]. 

Considering the correlation between elevated Rho-kinase activity in circulating white blood cells and a rise in angina frequency, as well as a worse prognosis in terms of cardiac events, the use of a Rho-kinase inhibitor shows potential as a technique to reduce the risk. Thus, intracoronary infusion of fasudil, a specific inhibitor of Rho-kinase, has successfully reduced coronary vasospasm and improved myocardial ischemia caused by spasms of the small blood vessels in the heart. However, there is currently no authorized oral Rho-kinase inhibitor. Although not extensively examined in relation to MINOCA, other indicators of CAS and CMD are equally significant based on the existing data. SuPAR serves as a reliable indicator of CMD in patients with non-obstructive CAD and may identify those who are at risk of experiencing long-term adverse effects. Cystatin C (CysC) demonstrated an independent association with the prevalence of CAS and had a prognostic potential for unfavorable outcomes. A different investigation discovered a direct relationship between the levels of serum CysC and the severity of coronary lesions in individuals with MINOCA. It was shown that elevated CysC levels independently increased the risk of negative outcomes in these patients [56,57,58].

According to the VERA trial, patients with symptomatic epicardial or microvascular coronary artery spasms do not experience improvement in their anginal symptoms after receiving add-on therapy with macitentan, a powerful ET-1 inhibitor. A randomized study by Reriani et al. found that Atrasentan, a selective ETA-receptor antagonist, significantly improved coronary microvascular endothelial function after 6 months of treatment, unlike the VERA study [59]. 

The Angina (PRIZE) experiment was a randomized, double-blind, placebo-controlled study conducted to uncover new genetic risk loci for CMD. The results suggest that dysregulation of ET-1 is involved, providing evidence for the potential use of genetic-based precision medicine to target oral ETA antagonist treatment in individuals with microvascular angina. Zibotentan is a molecule that has the potential to be the most ETA-selective of all orally active endothelin A (ETA) receptor antagonists. This makes it a good choice for microvascular angina [60]. In addition, a recent pilot study has indicated that administering a 10 mg dose of zibotentan has a beneficial effect on decreasing the frequency of angina in patients with refractory angina caused by the coronary slow-flow phenomenon [61].

In MINOCA patients, there was an elevated level of renin, suPAR, and IL-6 compared to healthy controls. The latter two markers are believed to be linked to worse cardiovascular outcomes [62]. High coronary tortuosity prevalence in spontaneous coronary artery disease (SCAD) patients is linked to recurrent SCAD, with TGF-β (transforming growth factor β) activity potentially impacting vascular tortuosity extent, according to a hypothesis. TGF-β has the potential to serve as an indicator of fibromuscular dysplasia (FMD) [49,63]. Eosinophils contribute to SCAD development by producing cytotoxic substances in response to inflammatory signals. Research on eosinophilic inflammation in SCAD has identified drugs that can reduce damage and facilitate healing by inhibiting chemotaxis, survival, and degranulation of eosinophils in their natural location [64]. 

Approximately 60% to 90% of people with angina and ANOCA have CMD as the underlying cause [5]. Recent research demonstrates an independent correlation between the presence of INOCA and a high SII (systemic immune-inflammation index) level. The SII value, in addition to the conventional, expensive approaches often used in INOCA prediction, can serve as an indicator [65].

Patients diagnosed with autoimmune illnesses (lupus, psoriasis, and rheumatoid arthritis) exhibit indications of reduced myocardial flow reserve (MFR), which cannot be accounted for by conventional risk factors for CAD. The WISE research found that women with INOCA who had a high IL-6 level were more likely to be hospitalized for HF and experience death from any cause within a period of 6 years [66,67].

A clinical trial involving autologous intracoronary CD34+ stem cell treatment in patients with INOCA showed promising results in improving angina symptoms, CFR, and overall quality of life after 6 months. CD34+ cells have the ability to undergo differentiation into endothelial cells, restoring microcirculation integrity [68].

The IMPROvE-CED study, which included patients with endothelium-dependent microvascular dysfunction, conducted another experiment to investigate CD34+ stem cell treatment. The trial had comparable outcomes to the previously stated trial, demonstrating substantial improvement in symptoms and angina class. Furthermore, the experiment demonstrated a rise in the proportion of ACh-mediated coronary blood flow (CBF) and a decrease in the daily use of sublingual nitroglycerin. Regenerative treatment with stem cells or gene therapy is a promising and innovative approach to treating CMD [69].

## 5. Integration of Multimodal Imaging

Both obstructive and non-obstructive forms of CAD may have extended stable intervals but can also suddenly become unstable [70]. The treatment for non-obstructive CAD varies greatly due to its diverse underlying causes, underscoring the importance of accurate diagnosis. Non-invasive imaging methods such as single-photon emission computed tomography (SPECT), positron emission tomography (PET), cardiac magnetic resonance (CMR), or coronary computed tomography angiography (CCTA) are crucial for detecting and evaluating non-obstructive CAD, providing both anatomical and functional insights. However, these non-invasive techniques cannot consistently detect conditions like microvascular spasm and coronary endothelial dysfunction, highlighting some limitations in their diagnostic capacity [71,72,73,74]. The ESC’s guidelines and the American College of Cardiology/American Heart Association ACC/AHA recommendations emphasize the importance of these imaging tests in diagnosing, treating, and assessing the risk of CAD. Ultimately, for a comprehensive evaluation, particularly of conditions like INOCA, invasive angiography remains necessary to assess endothelial-dependent dysfunction and other subtle coronary pathologies [2,70,75,76]. 

### 5.1. Coronary Computed Tomography Angiography

Coronary CT angiography is highly sensitive and accurate in detecting and ruling out CAD and abnormalities, though it has low specificity and positive predictive value. CCTA can evaluate myocardial perfusion by monitoring contrast flow from coronary vessels into the heart muscle at rest and post-adenosine injection, with decreased perfusion indicated by reduced contrast agent uptake. Despite its diagnostic benefits, including detailed plaque assessment and perfusion analysis, CCTA poses risks such as radiation exposure and contrast-induced nephropathy, particularly in patients with chronic renal disease. Technological advancements have significantly reduced radiation exposure. While CCTA is not currently supported for diagnosing MINOCA, it shows promise in identifying high-risk plaques and coronary inflammation. The WARRIOR study suggests a potential link between non-invasive CCTA and lower major adverse cardiovascular events (MACE) in women with suspected INOCA [77,78,79,80].

### 5.2. Cardiac Magnetic Resonance

Cardiac magnetic resonance imaging is crucial for evaluating suspected MINOCA, offering high diagnostic accuracy without ionizing radiation. CMR can distinguish between ischemic and non-ischemic heart muscle injuries using T2 and late gadolinium enhancement (LGE) sequences, providing a conclusive diagnosis in 65–99% of cases. Both the ESC and AHA endorse CMR for diagnosing MINOCA due to its ability to identify underlying causes of myocardial damage. Studies show that CMR can modify clinical diagnosis and treatment in over 50% and 32–42% of patients, respectively, especially when performed within two weeks of symptom onset. CMR’s ability to measure myocardial perfusion reserve (MPRI) helps in assessing CMD and predicting future cardiac events. Despite its benefits, CMR’s adoption is limited by cost, examination time, and contraindications in patients with certain conditions. Advancements in technology and techniques are needed to improve its accessibility and reduce scan durations [19,73,81,82,83,84,85].

### 5.3. Single-Photon Emission Computed Tomography and Positron Emission Tomography

SPECT and PET technologies use similar reconstruction methods to create heart images but differ in radiopharmaceuticals and imaging gear. SPECT and PET technologies are used to create heart images but differ in their radiopharmaceuticals and imaging equipment. Cardiac PET is highly reliable for assessing INOCA by measuring myocardial blood flow (MBF) and myocardial perfusion reserve (MPR), but its use is limited by availability and cost. PET can identify CMD and predict MACEs by evaluating MFR, with values less than 1.5 indicating CMD. SPECT, while showing a good long-term prognosis for patients with reversible ischemia and no significant CAD, also utilizes MBF and CFR as predictors of MACEs. Both modalities provide critical insights into CMD and associated risks, but PET remains the gold standard for non-invasive CMD diagnosis. Recent studies highlight the significant role of CMD in conditions like heart failure with preserved ejection fraction (HfpEF), suggesting that nearly 75% of HFpEF patients have underlying CMD [86,87,88,89].

In Table 2, imaging aspects of INOCA, ANOCA, and MINOCA, are presented.

### 5.4. Invasive Investigations

A comprehensive invasive evaluation involving angiography, physiological flow reserve measurements (like fractional flow reserve [FFR]), provocative testing, and CFR can effectively rule out myocardial ischemia as the primary cause of symptoms, facilitating appropriate non-cardiac examinations. Epicardial vasospasm is diagnosed by injecting ACh into the coronary artery, while microvascular dysfunction is evaluated by testing coronary reactivity to endothelium-independent and dependent dysfunctions. CFR assesses the impairment of blood flow, indicating the microcirculation’s ability to dilate blood vessels, especially when there is no epicardial stenosis [20,107].

For accurate diagnosis, invasive tests must distinguish between various pathophysiological mechanisms like spasm and microvascular resistance, using tools such as Doppler flow velocity or thermodilution. FFR evaluates flow-limiting obstructive CAD, while ACh-induced vasoconstriction diagnoses endothelial dysfunction. Comprehensive assessment identifies CMD using CFR and IMR measurements, and vasospastic responses via epicardial artery response to ACh [108,109].

The thermodilution-based index of microvascular resistance (T-IMR) is the most reliable approach among invasive techniques but has limitations due to its invasive nature. Non-invasive methods like PET and CMR, though effective, face challenges such as high costs and limited availability. Newly developed functional coronary angiography parameters, like a coronary angiography-derived index of microvascular resistance (caIMR), offer a simpler, faster procedure without the need for hyperemic medications [110,111,112].

Studies show that caIMR accurately measures microcirculatory resistance and has strong predictive value for adverse cardiovascular outcomes. Comprehensive evaluations incorporating these techniques improve the diagnosis and management of patients with suspected INOCA/MINOCA/ANOCA. Tailored medical therapies based on invasive coronary function testing, as evidenced by the CorMicA study, improve the quality of life in patients, highlighting the need for a pathophysiology-driven strategy with targeted therapeutics [27,113,114,115,116].

## 6. Limitations and Future Directions

Despite growing knowledge of the importance of properly examining individuals with angina who have undergone angiography but do not have obstructed coronary arteries, as well as new guideline recommendations, the real-world treatment and outcomes of such patients remain poorly understood. The MINOCA-BAT study is expected to provide results on this issue by 2025. Prognostic markers that have been validated in cases of classic MI should be further evaluated for their reliability in patients with myocardial infarction with MINOCA [117,118]. Additional research is necessary to confirm the effectiveness of the novel CMR approach (such as Strain-Encoded Magnetic Resonance) in accurately diagnosing MINOCA. There is a need for fast scanning methods in clinical practice to decrease scan durations and optimize efficiency and scanning capacity while maintaining diagnostic accuracy. It would be very useful to develop additional biomarkers that can accurately indicate the degree of microcirculatory function or damage in order to enhance the diagnosis of CMD. Also, there is a scarcity of research examining non-invasive imaging approaches for diagnosing CMD. Microvascular dysfunction in specific organs may indicate a larger issue, and studying diseases affecting small blood vessels in the brain, retina, kidney, lung, and heart could reveal common pathological pathways for new treatment approaches.

There are some limitations to this study. First, the complexities of non-obstructive CAD and its variants (ANOCA, INOCA, and MINOCA) mean that a comprehensive understanding is challenging to achieve through a narrative review alone. The causal mechanisms, particularly the role of microvascular dysfunction and its interaction with immunoinflammatory pathways, require further elucidation through controlled clinical trials and multi-center studies. Additionally, the review relies on the existing literature, which may have inherent biases or limitations in study design and population diversity. Another limitation is the lack of longitudinal data to firmly establish the long-term effectiveness of proposed diagnostic and therapeutic strategies. Lastly, while this review advocates for a shift in clinical practice, the actual implementation of these recommendations requires validation in diverse healthcare settings to assess feasibility and cost-effectiveness. 

## 7. Conclusions

This review highlights the prevalence and clinical impact of conditions such as ANOCA, INOCA, and MINOCA, which often go unrecognized due to the absence of obstructive lesions. As the pathophysiology involves microvascular dysfunction and increased coronary vasoreactivity, diagnostic and therapeutic approaches should address these underlying mechanisms in patients with nonobstructive CAD. The advocacy for a paradigm shift in treating these widespread conditions could potentially enhance patient outcomes by tailoring interventions to the specific vascular and inflammatory pathways involved.

## Figures and Tables

**Table 1:** Distribution of mechanisms in ANOCA, INOCA, and MINOCA.

Type	Mechanisms	Distribution of Mechanisms
**ANOCA**	Macrovascular dysfunction (epicardial coronary artery spasm) [15]. Coronary microvascular dysfunction (microvascular spasm, coronary slow flow, microvascular ischemia, impaired microvascular vasodilator response) [16].	N = 1196. Distribution: The endotypes were microvascular dysfunction in 24.5%, vasospastic angina microvascular in 25.7%, and vasospastic macrovascular angina in 25.4%. Nonspecific chest pain was present in 20.7% [11].
**INOCA**	Microvascular dysfunction [1]. Coronary vasospasm [17].	N = 1439. Distribution: The endotypes were microvascular dysfunction in 173 (12%), vasospastic angina in 478 (33.2%), combined microvascular and vasospastic angina in 268 (18.6%), and noncardiac chest pain in 520 (36.1%) [9].
**MINOCA**	Plaque disruption (plaque erosion or rupture) [1]. Coronary microvascular dysfunction, vasospasm, coronary thrombosis/embolism, spontaneous coronary artery dissection [7]. Endothelial dysfunction and heightened vasoreactivity [1].	N = 80. Distribution: epicardial spasm was detected in 24 (64.9%) patients and microvascular spasm in 13 (35.1%) patients [18]. N = 145. Plaque disruption was observed in 46.2% (67/145) by OCT. A nonischemic pattern of CMR abnormalities (myocarditis, takotsubo syndrome, or nonischemic cardiomyopathy) was present in 20.7% (24/116) [19].

ANOCA, angina with no obstructive coronary artery disease; CMR, Cardiac Magnetic Resonance; INOCA, ischemia with no obstructive coronary arteries; MINOCA, myocardial infarction with non-obstructive coronary arteries; OCT, Optical Coherence Tomography.

**Table 2:** Imaging aspects of ANOCA, INOCA, and MINOCA.

Type	Imaging Aspects
**INOCA**	Non-invasive CCTA testing has been associated with a decreased incidence of MACE in women with suspected INOCA [80]. The CMR MPR index is a reliable indicator of severe adverse cardiac events and has been linked to left ventricular diastolic function [8,90,91]. A cardiac PET measurement with an MRF below 1.5 indicates CMD and increases the risk of future cardiac events [30,92,93]. There is a direct correlation between hospitalization for HFpEF and a reduced MFR [94].
**ANOCA**	-
**MINOCA**	CCTA may identify vulnerable plaques that are normally undetected by invasive coronary angiography [79]. CMR can provide insight into underlying causes, potentially influencing the therapy method further [14,73,83,95,96,97,98]. Extent of LGE, along with elevated T2 mapping values, serve as robust indicators of unfavorable outcomes [99]. Quantitative stress CMR perfusion mapping can distinguish CMD from obstructive CAD [100]. Both stress MBF and MPR evaluated by PET yielded predictions of MACEs [87]. Decreased MFR measured by cardiac PET is predictive of MACE in female patients [30].
**VSA**	-
**CMD**	CFR assessment and the myocardial perfusion reserve index are much better at predicting the risk of MACE than LGE and ischemia alone [101]. Quantitative CMR techniques offer improved diagnostic capabilities for MVD, accurate assessment of CAD severity, and quick patient risk stratification [8,102,103,104]. The MPR value is associated with an increased risk for death and MACE [90,103]. PET-derived CFR is directly linked to diastolic dysfunction and cardiovascular adverse events [105]. The low MPR PET-derived value can accurately predict the long-term likelihood of significant adverse cardiovascular events [106].

ANOCA = angina with no obstructive coronary artery disease; CAD = coronary artery disease; CCTA = coronary computed tomography angiography; CFR = coronary flow reserve; CMR = cardiac magnetic resonance; CMD = coronary microvascular dysfunction; INOCA = ischemia with no obstructive coronary arteries; HF = heart failure; HFpEF = heart failure with preserved ejection fraction; LGE = late gadolinium enhancement; MACE = major adverse cardiac events; MINOCA = myocardial infarction with non-obstructive coronary arteries; MBF = myocardial blood flow; MFR = myocardial flow reserve; MPR = myocardial perfusion reserve; MVD = microvascular disease; PET = positron emission tomography; VSA = vasospastic angina.
